# Validation of a polygenic risk score for dementia in black and white individuals

**DOI:** 10.1002/brb3.248

**Published:** 2014-07-18

**Authors:** Jessica R Marden, Stefan Walter, Eric J Tchetgen Tchetgen, Ichiro Kawachi, M Maria Glymour

**Affiliations:** 1 Department of Social and Behavioral Sciences, Harvard School of Public Health677 Huntington Ave, Boston, Massachusetts, 02115; 2 Department of Epidemiology and Biostatistics, University of California at San FranciscoSan Francisco, California; 3 Department of Biostatistics, Harvard School of Public Health677 Huntington Ave, Boston, Massachusetts, 02115; 4 Department of Epidemiology, Harvard School of Public Health677 Huntington Ave, Boston, Massachusetts, 02115

**Keywords:** Dementia, genetics, race

## Abstract

**Objective:**

To determine whether a polygenic risk score for Alzheimer's disease (AD) predicts dementia probability and memory functioning in non-Hispanic black (NHB) and non-Hispanic white (NHW) participants from a sample not used in previous genome-wide association studies.

**Methods:**

Non-Hispanic white and NHB Health and Retirement Study (HRS) participants provided genetic information and either a composite memory score (*n* = 10,401) or a dementia probability score (*n* = 7690). Dementia probability score was estimated for participants' age 65+ from 2006 to 2010, while memory score was available for participants age 50+. We calculated AD genetic risk scores (AD-GRS) based on 10 polymorphisms confirmed to predict AD, weighting alleles by beta coefficients reported in AlzGene meta-analyses. We used pooled logistic regression to estimate the association of the AD-GRS with dementia probability and generalized linear models to estimate its effect on memory score.

**Results:**

Each 0.10 unit change in the AD-GRS was associated with larger relative effects on dementia among NHW aged 65+ (OR = 2.22; 95% CI: 1.79, 2.74; *P* < 0.001) than NHB (OR=1.33; 95% CI: 1.00, 1.77; *P* = 0.047), although additive effect estimates were similar. Each 0.10 unit change in the AD-GRS was associated with a −0.07 (95% CI: −0.09, −0.05; *P* < 0.001) SD difference in memory score among NHW aged 50+, but no significant differences among NHB (*β* = −0.01; 95% CI: −0.04, 0.01; *P* = 0.546). [Correction added on 29 July 2014, after first online publication: confidence intervalshave been amended.] The estimated effect of the GRS was significantly smaller among NHB than NHW (*P* < 0.05) for both outcomes.

**Conclusion:**

This analysis provides evidence for differential relative effects of the GRS on dementia probability and memory score among NHW and NHB in a new, national data set.

## Introduction

Previous candidate gene and genome wide association studies (GWAS) have implicated several genetic loci in the development of Alzheimer's disease (AD), primarily in European ancestry samples. Apart from APOE, effect sizes for individual alleles are generally small, but when combined into a polygenic risk score, these loci may have large joint effects on dementia risk (Okuizumi and Tsuji 1998).

There is an urgent need to explore whether these genetic associations are generalizable to other populations and consistent across racial groups. Evidence regarding the association between the APOE *ε*4 allele and AD among nonwhite populations has been mixed, but the majority of studies report weaker associations among ethnic minorities than among non-Hispanic whites (NHW) (Hendrie et al. 1995; Tang et al. 1996, 1998; Maestre et al. 2004; Osuntokun et al. 2004; Bekris et al. 2010; Barnes et al. 2013). Limited research has been conducted examining associations between other genetic loci and AD in blacks or other racial groups (Nussbaum 2013).

Survival bias is a potential problem in analyses seeking to uncover genetic risk factors for AD, and it may explain different findings across racial groups. Analyses in samples of older adults inherently exclude people who died before old age. Selective survival can induce an underestimation of the genetic effects on dementia, especially in populations with high mortality rates at younger ages (Glymour 2007).

We report here the associations of a 10-gene polygenic risk score (AD-GRS) with memory function and dementia probability in a large, public use data set, the Health and Retirement Study (HRS). Since previous GWAS of AD have not been conducted in HRS, observed associations provide new evidence on the generalizability of previously reported predictive loci. HRS is a nationally representative sample, with sufficient representation of non-Hispanic blacks (NHB) to permit separate investigation of the AD-GRS among NHW and NHB sample members. We report the findings for the AD-GRS both including and excluding APOE. We hypothesized that there would be a similar additive effect of the AD-GRS on dementia probability and memory score in NHW and NHB, regardless of the inclusion of the APOE gene or age at assessment.

## Methods

### Study population

Health and Retirement Study is a nationally representative cohort study initiated in 1992 with enrolments in 1992, 1993, and 1998. The target population was all adults in the contiguous United States born between 1931 and 1941 living in a household (Hauser and Willis 2004). Biennial interviews (or proxy interviews for decedent participants) are available through 2010. Details of the study are provided elsewhere (Heeringa and Connor 1995; Juster and Suzman 1995; Ofstedal et al. 2005). Original survey response rates varied across enrolment cohorts from 70% to 82%, and retention rates through 2008 ranged from 86% to 91% (Health and Retirement Study 2011).

This paper uses a separate sample genetic instrumental variable (IV) methodology in this prospective cohort. Our analyses used a sub-sample with genetic data collected in 2006 or 2008. To maximize statistical power, we used repeated observations (up to 3) on the same individuals. From 12,123 HRS participants with genetic data, we restricted to 10,444 (86.2%) who were aged 50+ in 2006, contributed at least one cognitive assessment in 2006, 2008, or 2010, and who self-identified as NHW or NHB. The final analytic samples differed for our two outcomes, dementia probability and composite memory. In models predicting dementia probability, from 31,332 possible observations (10,444 participants by three time points), we excluded observations for participants who were under 65 (9792, 31.1%), missing dementia probability due to nonresponse (695, 2.2%) or had died by that interview year (1063, 3.4%); the remaining 7690 individuals contributed 19,782 observations over the follow-up period of up to 4.0 years. In the models predicting memory score, from the same 31,332 possible observations we excluded observations missing memory score due to nonresponse (1261, 4.0%) or death (1168, 3.7%); the remaining 10,401 individuals contributed 29,062 observations on memory score.

Health and Retirement Study is approved by the University of Michigan Health Sciences Human Subjects Committee and all participants provided written informed consent. The Harvard School of Public Health Human Subjects Committee determined the current analyses exempt. The procedures followed were in accordance with the ethical standards of with the Helsinki Declaration of 1975, as revised in 2008.

### Dementia probability outcomes

Dementia probability score was estimated based on word list memory, telephone interview for cognitive status (TICS), and the Informant Questionnaire for Cognitive Decline (IQCODE) and was previously shown to predict DSM-IV defined dementia, with a *c*-statistic of 94.3% (Wu et al. 2012). For individuals too impaired to directly participate in memory assessments, proxy informants, typically spouses, were asked to assess the participants' memory on a 5-item Likert scale and completed a 16-item version of the IQCODE (Jorm 1994; Jorm et al. 2000). We used a previously developed dementia probability score combining proxy and direct memory assessments for longitudinal analyses (Wu et al. 2012). The composite score algorithm was developed in an 856-subject subsample who participated in a comprehensive neuropsychological battery as part of the Aging, Demographics, and Memory Study (Langa et al. 2005; Plassman et al. 2011). Further details of development and validation of the memory score are available from the authors. This score was only calculated for HRS respondents who were 65 years or older because the TICS was only routinely assessed in this age group. The score is continuous, changes over time within an individual, and has a possible range from 0 (no chance of dementia) to 1 (certain to have dementia). For example, a score of 0.2 indicates a 20% probability the individual would meet DSM-IV dementia criteria.

### Memory score outcomes

Memory was assessed by immediate and delayed recall of a 10-word list plus the proxy assessments for severely impaired individuals. The validity and reliability of these measures have been documented elsewhere (Ofstedal et al. 2005; Wu et al. 2012). As described above, the composite score algorithm was applied in order to calculate this memory score. We standardized the memory score by dividing each score by the 1998 standard deviation so that every unit change in memory score corresponds to approximately one standard deviation in the population.

### Genotyping

The Health and Retirement Study genetic data are sponsored by the National Institute on Aging (grant numbers U01AG009740, RC2AG036495, and RC4AG039029) and was conducted by the University of Michigan. In 2006 and 2008, HRS invited participants to provide DNA samples. Eligible respondents were consented and provided saliva via a mouthwash technique (average age at DNA collection: 68 years). Genotyping was completed on the Illumina Omni-2.5 chip platform and imputed using the 1000G phase 1 reference panel. Genetic information for the first 12,507 participants was filed with the Database for Genotypes and Phenotypes (dbGaP, study accession number: phs000428.v1.p1) in April 2012. Principle components were used to identify and remove population outliers. Exact information on the QC procedures applied is available via HRS and dbGaP (Health and Retirement Study 2010).

### Exposure to genetic risk of dementia

Two SNPs (rs7412 and rs429358) are commonly used to identify APOE *ε*4 variants, which conveys substantially increased risk of dementia (Whalley et al. 2006; Bekris et al. 2010). Several other genetic loci have been confirmed as genome-wide significant predictors of AD, with meta-analyzed odds ratios (ORs) reported in the AlzGene online database (Bertram et al. 2007). After APOE, the 9 loci with the most significant *P*-values as of January 13^th^, 2013 were: BIN1, CLU, ABCA7, CR1, PICALM, MS4A6A, CD33, MS4A4E, and CD2AP (Table S1 for ORs) (Bertram et al. 2007). We calculated the AD-GRS by multiplying each individual's risk allele count for each locus by the reported beta coefficient for that polymorphism from AlzGene and summing the product for all 10 loci, noting that the betas are generally from logistic regression models and therefore correspond with the natural log of the OR (eq. 2014). This step essentially weighted each polymorphism in proportion to its anticipated effect on dementia risk. Next, to convert to the odds of dementia for each individual, we exponentiated the weighted allele sum and multiplied the resulting value by 0.1, the estimated dementia prevalence in the sample (eq. 2013), and converted odds into probabilities (eq. 2010).


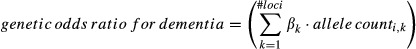
(1)


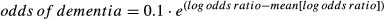
(2)


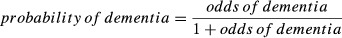
(3)

The AD-GRS can be interpreted as the probability of dementia predicted by the 10 alleles, given the strength of the associations estimated in previously published GWAS and AlzGene meta-analyses. We also calculated an alternative AD-GRS excluding APOE.

### Race

We used self-reported race (“What race do you consider yourself to be: White, Black or African American, American Indian, Alaska Native, Asian, Native Hawaiian, Pacific Islander, or something else?”), and included only NHW and NHB due to small sample sizes in other racial groups and complexity of Hispanics' genetic backgrounds.

### Other covariates

All models were adjusted for sex, age at dementia/memory assessment, and interview year. We did not include the eigenvectors to control for population stratification because we were interested in the effect of self-reported race. Results estimated with controlling for the eigenvectors are included in Tables S2–S4 and are not qualitatively different from results estimated without eigenvectors (Weir 2012).

### Statistical analysis

We used pooled logistic regression to estimate the association of the AD-GRS with dementia probability assessed from 2006 to 2010 and generalized linear models to estimate the effect of the AD-GRS on memory score, using clustered standard errors to account for repeated measures on the same individual. We show results stratified by race (NHW and NHB) and also estimated in race-pooled models with interactions to test whether effect estimates differed significantly for NHW and NHB. Next, each of these models was replicated using the alternative AD-GRS which excluded APOE. Additionally, we tested whether APOE and the ABCA7 SNP recently reported to predict dementia among blacks (rs115550680)(Reitz et al. 2013) independently predicted dementia in our sample. Finally, we estimated age- and gender-stratified models for both NHW and NHB samples and tested an age by AD-GRS interaction to assess the plausibility of substantial survival bias. A *P*-value of 0.05 or lower was considered to be statistically significant.

## Results

**Figure 1:**
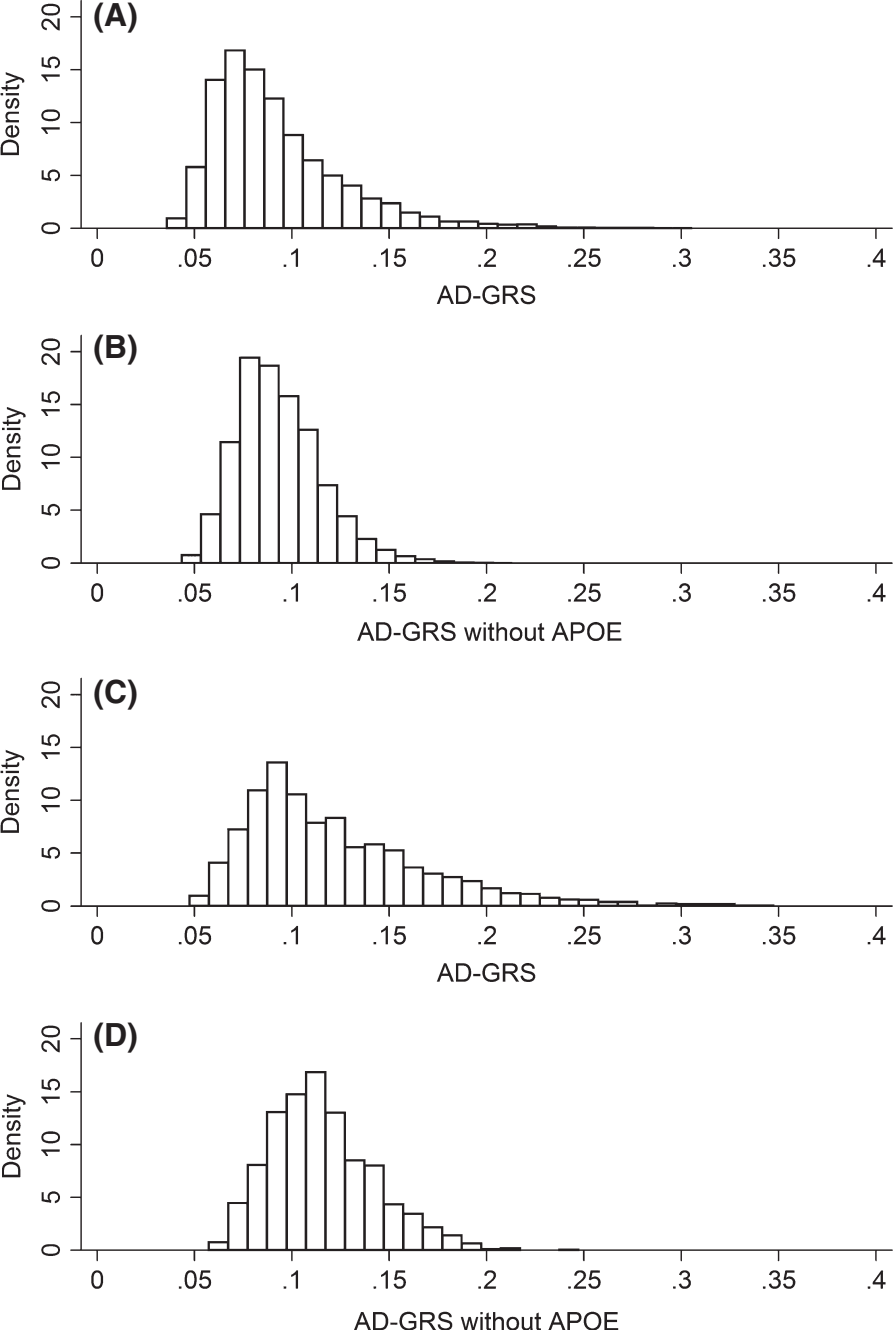
Histograms of the distribution of the genetic risk score with and without APOE among non-Hispanic whites and blacks. (A) Distribution of the AD-GRS including APOE among NHW (mean = 0.09, SD = 0.03). (B) Distribution of the AD-GRS excluding APOE among NHW (mean = 0.09, SD = 0.02). (C) Distribution of the AD-GRS including APOE among NHB (mean = 0.12, SD = 0.05). (D) Distribution of the AD-GRS excluding APOE among NHB (mean = 0.12, SD = 0.03).

Characteristics from the first wave at which each individual contributed an observation are shown for the 7690 individuals used in the dementia probability models and the 10,401 individuals used in the memory score models (Tables 1 and 2). The AD-GRS was centered around a 0.09 probability of dementia in NHW and a 0.12 probability in NHB (Fig. 1).

**Table 1:** Characteristics of the dementia probability sample from the first wave that each individual contributed an observation, health and retirement study 2006–2010

	All	NHW	NHB
			
	*N* = 7690	*N* = 6675	*N* = 1015
Demographics			
Male, No. (%)	3273	2890	383
	(42.6%)	(43.3%)	(37.7%)
Age, mean (SD), years	72.82	74.00	71.63
	(6.87)	(7.08)	(6.62)
Education, mean (SD), years	12.75	12.97	11.31
	(2.74)	(2.56)	(3.35)
Genetic Risk Score			
GRS, mean (SD)	0.096	0.092	0.122
	(0.037)	(0.034)	(0.048)
GRS no APOE, mean (SD)	0.096	0.093	0.115
	(0.024)	(0.022)	(0.026)
Cognitive Outcome			
Dementia Probability, mean (SD)	0.042	0.037	0.077
	(0.130)	(0.119)	(0.182)
Health Conditions and Behaviors			
Ever had a stroke, No. (%)	699	589	110
	(9.1%)	(8.8%)	(10.8%)
Ever had diabetes, No. (%)	1530	1197	333
	(19.9%)	(18.0%)	(32.8%)
Ever had hypertension, No. (%)	4692	3907	785
	(61.1%)	(58.6%)	(77.42%)
Ever had heart problems, No. (%)	2257	1996	261
	(29.4%)	(29.9%)	(25.7%)
Ever smoked, No. (%)	4440	3858	582
	(58.0%)	(58.1%)	(57.7%)
Total Cholesterol, mean (SD)^1^	198.59	198.78	197.28
	(41.67)	(41.87)	(40.28)

1 These data were not available for everyone in the sample because they were obtained from a biomarker sub-sample of the HRS. These means and standard deviations are based on 6729 total participants (5875 NHW and 854 NHB) who were included in this subsample.

**Table 2:** Characteristics of the memory score sample from the first wave that each individual contributed an observation, health, and retirement study 2006–2010

	All	NHW			NHB		
			
		All	Age 50–64	Age 65+	All	Age 50–64	Age 65+
							
	*N* = 10,401	*N* = 8942	*N* = 3319	*N* = 6630	*N* = 1459	*N* = 642	*N* = 993
Demographics							
Male, No. (%)	4281	3759	1281	2859	522	205	372
	(41.5%)	(42.0%)	(38.6%)	(43.1%)	(35.8%)	(31.9%)	(37.5%)
Age, mean (SD), years	67.87	68.16	57.70	73.01	66.12	57.39	71.68
	(10.00)	(10.04)	(4.16)	(7.07)	(9.60)	(4.14)	(6.64)
Education, mean (SD), years	12.99	13.20	13.71	12.97	11.70	12.59	11.21
	(2.68)	(2.52)	(2.33)	(2.56)	(3.19)	(2.57)	(3.37)
Genetic Risk Score							
GRS, mean (SD)	0.097	0.093	0.094	0.092	0.123	0.125	0.122
	(0.038)	(0.035)	(0.036)	(0.034)	(0.048)	(0.049)	(0.048)
GRS no APOE (mean, SD)	0.097	0.093	0.094	0.093	0.115	0.116	0.116
	(0.024)	(0.022)	(0.022)	(0.022)	(0.027)	(0.027)	(0.026)
Cognitive Outcome							
Memory Score, mean (SD)	1.013	1.077	1.365	0.956	0.663	0.989	0.476
	(0.464)	(0.437)	(0.171)	(0.444)	(0.472)	(0.194)	(0.461)
Health Conditions and Behaviors							
Ever had a stroke, No. (%)	775	633	107	587	142	50	110
	(7.5%)	(7.1%)	(3.2%)	(8.9%)	(9.7%)	(7.8%)	(11.1%)
Ever had diabetes, No. (%)	1843	1428	436	1192	415	155	327
	(17.7%)	(16.0%)	(13.1%)	(18.0%)	(28.5%)	(24.2%)	(33.0%)
Ever had hypertension, No. (%)	5720	4683	1373	3883	1037	408	774
	(55.1%)	(52.4%)	(41.4%)	(58.7%)	(71.1%)	(63.6%)	(78.0%)
Ever had heart problems, No. (%)	2564	2241	479	1989	323	105	258
	(24.7%)	(25.1%)	(14.4%)	(30.0%)	(22.2%)	(16.4%)	(26.0%)
Ever smoked, No. (%)	5908	5060	1856	3817	848	384	571
	(57.0%)	(56.8%)	(56.1%)	(57.8%)	(58.4%)	(59.9%)	(57.9%)
Total Cholesterol, mean (SD)^1^	201.27	201.50	209.07	198.68	199.76	205.57	196.95
	(42.03)	(42.14)	(41.94)	(41.90)	(41.28)	(42.30)	(40.52)

1 These data were not available for everyone in the sample because they were obtained from a biomarker sub-sample of the HRS. These means and standard deviations are based on 9164 total participants (7934 NHW and 1230 NHB) who were included in this subsample.

In a pooled model including both NHW and NHB, each 0.10 unit increase in the AD–GRS was associated with an OR of 1.90 (95% CI: 1.59, 2.27; *P* < 0.001) for dementia risk (Table 3). Among NHW, the AD-GRS was associated with an OR of 2.22 (95% CI: 1.79, 2.74; *P* < 0.001) for dementia risk, compared to an OR of 1.33 (95% CI: 1.00, 1.77; *P* = 0.047) among NHB. The interaction term between race and the AD-GRS was statistically significant (*P* = 0.014), indicating a weaker association between the AD-GRS and dementia among NHB compared to NHW.

**Table 3:** Pooled logistic models: odds ratios for dementia by genetic risk score stratified by race

	All (Race Pooled) *N* = 7690 OR (95% CI)		NHW (Race Stratified) *N* = 6675 OR (95% CI)		NHB (Race Stratified) *N* = 1015 OR (95% CI)		All (Race Interaction)*N* = 7690 OR (95% CI)	
				
	Model A^1^ (AD-GRS)	Model B^2^(AD-GRS ex APOE)	Model A^1^ (AD-GRS)	Model B^2^ (AD-GRS ex APOE)	Model A^1^ (AD-GRS)	Model B^2^ (AD-GRS ex APOE)	Model A^1^ (AD-GRS)	Model B^2^ (AD-GRS ex APOE)
GRS (0.1 increase)	1.90 (1.59, 2.27)**		2.22 (1.79, 2.74)**		1.33 (1.00, 1.77)*		2.19 (1.77, 2.69)**	
GRS (no APOE) (0.1 increase)		1.46 (1.11, 1.93)*		1.35 (0.98, 1.88)		1.72 (1.00, 2.96)		1.35 (0.98, 1.86)
Black	1.81 (1.50, 2.18)**	2.02 (1.68, 2.43)**					3.09 (1.95, 4.90)**	1.44 (0.68, 3.02)
GRS*Black							0.63 (0.44, 0.91)*	
GRS (no APOE) *Black								1.36 (0.72, 2.59)

All models are additionally adjusted for: age (linear), gender, and year of assessment.

* *P*-value < 0.05;

** *P*-value < 0.001.

1 Model A uses the regular 10 locus AD-GRS as a predictor.

2 Model B uses the alternative 9 locus AD-GRS excluding APOE as a predictor.

Dementia prevalence was higher among NHB than NHW, so the different relative effects are consistent with similar absolute effects. In absolute terms, these models predict that a NHW male age 70 with an AD-GRS of 0.1 (the mean) has a 1.10% probability of dementia. A similar man with a 0.137 AD-GRS (1 SD higher) has a 1.50% chance of dementia (a 0.4% point increase). In comparison, an NHB male age 70 with an AD-GRS of 0.1 has a 3.30% probability of dementia. A similar NHB man with a 0.137 AD-GRS has a 3.70% chance of dementia (a 0.4 percentage point increase). At average AD-GRS scores, an SD increase was associated with approximately a 0.4% point change in dementia probability for both races.

In a pooled model, each 0.10 unit change in the AD-GRS was associated with −0.06 (95% CI: −0.07, −0.04; *P* < 0.001) SD difference in memory score (Table 4). In the race-stratified models, the AD-GRS was associated with a −0.07 (95% CI: −0.09, −0.06; *P* < 0.001) SD difference in memory score in NHW and a −0.01 (95% CI: −0.03, 0.02; *P* = 0.546) SD difference in memory score in NHB (Table 4). There was evidence of a difference in the effect of the AD-GRS on memory score by race (*P* < 0.001 for interaction), with large adverse effects in NHW but an estimate of approximately zero among NHB.

**Table 4:** Generalized linear models: regression coefficients for memory score by genetic risk score stratified by race

	All (Race Pooled) *N* = 10,401 *β* (95% CI)		NHW (Race Stratified) *N* = 8942 *β* (95% CI)		NHB(Race Stratified) *N* = 1459 *β* (95% CI)		All(Race Interaction) *N* = 10,401*β* (95% CI)	
				
	Model A^1^(AD-GRS)	Model B^2^(AD-GRS ex APOE)	Model A^1^(AD-GRS)	Model B^2^(AD-GRS ex APOE)	Model A^1^(AD-GRS)	Model B^2^(AD-GRS ex APOE)	Model A^1^(AD-GRS)	Model B^2^(AD-GRS ex APOE)
GRS (0.1 increase)	−0.06 (−0.07, −0.04)**		−0.07 (−0.09, −0.05)**		−0.01 (−0.04, 0.01)		−0.07 (−0.09, −0.05)**	
GRS (no APOE) (0.1 increase)		−0.03 (−0.05, −0.01)*		−0.03 (−0.06, −0.01)*		−0.01 (−0.06, 0.04)		−0.04 (−0.06, −0.01)*
Black	−0.49 (−0.50, −0.47)**	−0.50 (−0.51, −0.48)**					−0.57 (−0.61, −0.53)**	−0.54 (−0.60, −0.47)**
GRS*Black							0.07 (0.04, 0.10)**	
GRS (no APOE) *Black								0.03 (−0.02, 0.09)

All models are additionally adjusted for: age (linear), gender, and year of assessment.

* *P*-value < 0.05;

** *P*-value < 0.001.

1 Model A uses the regular 10 locus AD-GRS as a predictor.

2 Model B uses the alternative 9 locus AD-GRS excluding APOE as a predictor.

In the pooled model using the alternative AD-GRS excluding APOE, the AD-GRS was associated with an OR of 1.46 (95% CI: 1.11, 1.93; *P* = 0.008) for dementia risk. Among NHW, the modified AD-GRS was associated with an OR of 1.35 (95% CI: 0.98, 1.88; *P* = 0.068) for dementia risk, compared to an OR of 1.72 (95% CI: 1.00, 2.96; *P* = 0.051) among NHB (Table 3) and there was no evidence that the ORs were significantly different for NHW versus NHB (*P* for interaction = 0.347).

In the pooled models predicting memory score, each 0.10 unit change in the AD-GRS excluding APOE was associated with a −0.03 (95% CI: −0.05, −0.01; *P* = 0.018) SD difference in memory score. In the race-stratified models, the AD-GRS was associated with a −0.03 (95% CI: −0.06, −0.01; *P* = 0.012) SD difference in memory score in NHW and a −0.01 (95% CI: −0.06, 0.04; *P* = 0.805) SD difference in memory score among NHB. The difference in point estimates was not statistically significant (*P* for interaction = 0.240).

When examining quintiles of the AD-GRS, the AD-GRS excluding APOE showed an approximately dose response association with dementia and memory. Using the GRS with APOE, there is a spike in risk among people who carry APOE, as would be expected given the much larger effect of APOE than any other locus. Having at least one APOE-*ε*4 allele was associated with an OR of dementia probability of 1.89 (95% CI: 1.53, 2.21; *P* < 0.001) in NHW and 1.30 (95% CI: 0.92, 1.85; *P* = 0.140) in NHB. In the memory score models, APOE was associated with a −0.06 (95% CI: −0.07, −0.04; *P* < 0.001) SD difference in memory score in NHW and a −0.02 (95% CI: −0.05, 0.002; *P* = 0.075) SD difference in NHB. These results are consistent with our above findings that APOE is less predictive of dementia and memory among NHB compared to NHW. ABCA7 SNP rs115550680, recently reported as associated with AD in blacks by Reitz et al., (Reitz et al. 2013) was not associated with dementia probability (OR = 0.99, 95% CI: 0.57, 1.70) in our NHB sample.

We estimated age-stratified models of memory score outcomes to evaluate possible survival bias (Table 5). Age-stratified models could not be estimated for the dementia probability sample, because dementia probability was only assessed for people aged 65+. Among NHW, the AD-GRS including APOE was associated with worse memory score for people aged 65+ but not people aged 50–64 (*P* < 0.001 for interaction). Among NHB, the AD-GRS including APOE was not associated with memory score for either age group, although the point estimate was suggestive in older NHB (*P* = 0.585 for interaction). Age-stratified models using the AD-GRS excluding APOE came to the same qualitative conclusion: evidence for a difference in the association between the AD-GRS and memory across age groups in NHW, but not in NHB.

**Table 5:** Age-stratified models: regression coefficients for memory score by genetic risk score stratified by race and age

	NHW						NHB					
		
	All (*N* = 8942)		Age 50–64 (*N* = 3319)		Age 65+ (*N* = 6630)		All (*N* = 1459)		Age 50–64 (*N* = 642)		Age 65+ (*N* = 993)	
						
	*β* (95% CI)		*β* (95% CI)		*β* (95% CI)		*β* (95% CI)		*β* (95% CI)		*β* (95% CI)	
						
	Model A^1^ (AD-GRS)	Model B^2^ (AD-GRS ex APOE)	Model A^1^ (AD-GRS)	Model B^2^ (AD-GRS ex APOE)	Model A^1^ (AD-GRS)	Model B^2^ (AD-GRS ex APOE)	Model A^1^ (AD-GRS)	Model B^2^ (AD-GRS ex APOE)	Model A^1^ (AD-GRS)	Model B^2^ (AD-GRS ex APOE)	Model A^1^ (AD-GRS)	Model B^2^ (AD-GRS ex APOE)
GRS (0.1 increase)	−0.02 (−0.04, −0.01)*		0.001 (−0.01, 0.01)		−0.10 (−0.13, −0.08)**		−0.01 (−0.03, 0.02)		−0.001 (−0.02, 0.02)		−0.03 (−0.06, 0.01)	
GRS (no APOE) (0.1 increase)		0.04 (0.01, 0.06)*		−0.01 (−0.02, 0.01)		−0.04 (−0.07, −0.01)*		−0.001 (−0.05, 0.05)		0.02 (−0.01, 0.05)		−0.04 (−0.11, 0.03)
Age 65+	0.27 (0.24, 0.30)**	0.30 (0.28, 0.33)**					0.18 (0.12, 0.24)**	0.19 (0.13, 0.25)**				
GRS*Age 65+	−0.08 (−0.10, −0.05)**						−0.01 (−0.05, 0.03)					
GRS (no APOE) *Age 65+		−0.11 (−0.14, −0.08)**						−0.02 (−0.05, 0.02)				

All models are additionally adjusted for: age (linear), gender, and year of assessment.

* *P*-value < 0.05;

** *P*-value < 0.001.

1 Model A uses the regular 10 locus AD-GRS as a predictor.

2 Model B uses the alternative 9 locus AD-GRS excluding APOE as a predictor.

There was no evidence that the mean AD-GRS differed by age groups (age 50–65 vs. 65+). The mean AD-GRS in both age groups was 0.09 for NHW and 0.12 for NHB. The APOE *ε*4 allele frequency was similar for NHW in each age group (0.29 for age 50–65, 0.28 for age 65+). However, it differed slightly by age for NHB (0.45 for age 50–65, 0.38 for age 65+).

Finally, we estimated gender-stratified models to explore whether the association between the AD-GRS and either dementia or memory score differed by gender. Among NHW, there was no evidence for differential effects by gender for either dementia (*P* = 0.757 for interaction of AD-GRS and gender) or memory (*P* = 0.670 for interaction of AD-GRS and gender). Similarly, among NHB, the gender-by-AD-GRS interaction terms for dementia (*P* = 0.174) and memory (*P* = 0.304) were not statistically significant. Overall, we did not find any evidence for differences between males and females within either racial group. Conversely, estimates obtained using the AD-GRS excluding APOE found all groups to be similar except NHB males and females for the dementia probability outcome (*P* = 0.047 for interaction of AD-GRS excluding APOE and gender). Among NHB males, the AD-GRS excluding APOE was associated with an OR of 3.60 (95% CI: 1.62, 8.00; *P* < 0.001) for dementia risk compared to an OR of 1.32 (95% CI: 0.66, 2.66; *P* = 0.436) among NHB females. However, there was no evidence for an APOE*gender interaction in our sample. Please see Tables S5 and S6 for complete gender-statified results.

## Discussion

Our results suggest the AD-GRS predicts dementia risk among both NHW and NHB. However, our findings suggest a difference in the relative effect of the AD-GRS on dementia probability and memory score by race, providing evidence against our original hypothesis. The AD-GRS including APOE had larger relative effects on dementia in NHW than in NHB, although absolute effects were similar. The AD-GRS also had significantly smaller relative effects on memory among NHB than NHW. However, a modified AD-GRS without APOE was associated with slightly larger effects on dementia probability for NHB than NHW. The difference in the magnitude of the OR for the AD-GRS with and without APOE in NHB predicting dementia probability (1.33–1.72) supports the hypothesis that APOE is not as strong a predictor of dementia in NHB as in NHW. The strong association of the AD-GRS without APOE with dementia probability in NHB is striking because the beta coefficients used to create the AD-GRS were largely drawn from studies of white populations.

The strength of any specific gene–disease association depends on the distribution of other causes of disease in the population (Rothman and Poole 1988). Thus, the relative impact of a gene such as APOE may be reduced among NHB if other causes of dementia are more common, or if APOE is causative only in the additional presence of other dementia risk factors that are uncommon in blacks. These findings therefore highlight the importance of identifying possible genetic and social modifiers of APOE effects to explain the divergent effect estimates for NHB and NHW.

This large, diverse data set with genetic information and repeated cognitive measures is uniquely suited for this research question. However, it has limitations. We have no information on gene expression or epigenetic modifications (Bird 2007; Zhang and Meaney 2010). Gene expression patterns could explain differences in dementia and/or memory in people with similar gene frequencies. Further, HRS does not have a clinical dementia diagnosis or assessments of many important domains of cognitive function and available measures likely have substantial measurement error. Additionally, the GRS is AD-based but the ascertainment of dementia is not sub-type specific (i.e., we don't know if demented patients have AD, or other types of dementia). Using both measures of memory function and dementia probability proved beneficial since we were able to compare results to check for consistency.

One strength of this study is the calculation of additive effect estimates in addition to the relative estimates. Many studies do not report estimates on the absolute scale since logistic regression models, commonly used due to the often-dichotomous nature of dementia outcomes, provide ORs – measures on the relative scale (Khoury et al. 1989). Since dementia is more common in NHB than NHW, this may be misleading. The relative estimates obtained in our study were smaller among NHB, but the absolute estimates were very similar to NHW and NHB. Therefore, it may be that NHB have differential exposure to other dementia risk factors, rendering the relative association between APOE and dementia smaller compared to the magnitude in NHW.

Additionally, we directly assessed the likely role of survival bias. Average AD-GRS was similar across age groups; differences in the relationship between the AD-GRS and memory in the age-stratified models are therefore probably not due to survival bias. Instead, these differences likely arise because between-individual memory differences before 65 years of age are mostly due to nondementia-related factors (e.g. socioeconomic status), whereas after 65 years of age memory changes are more likely to reflect dementia risk. However, survival bias remains a possibility in studies seeking to identify genetic determinants of chronic illnesses and thus should be assessed as a potential threat to validity in these cases. In particular, the lower prevalence of APOE *ε*4 in older blacks is especially important to evaluate: APOE is generally related to mortality and, if this effect is stronger in blacks than whites, it could attenuate the estimated effects of APOE in older blacks.

Finally, we estimated gender-stratified models to evaluate the potential for differential effects of the AD-GRS on dementia and memory among men and women. We found some evidence that the AD-GRS excluding APOE is more predictive of dementia among NHB males compared to NHB females. A recent study by Altmann et al. found that APOE *ε*4 confers greater AD risk in women (Altmann et al. 2014). One interpretation of our results could be consistent with Altmann's general findings: when APOE is removed from the AD-GRS, women's odds ratio for dementia decreases because APOE was the main driver of the association. However, for men, other genes may play more of a role in their dementia risk profile. One striking difference between our findings and Altmann's is that Altmann's results came from a sample that was approximately 80% NHW, while our only significant gender differences occurred in our NHB subsample. Further, we found no evidence of APOE*gender interaction in either NHW or NHB in our sample, suggesting potential alternative explanations for our gender-stratified results. In order to understand the differences in AD genetic risk profiles by race and gender, more studies with larger samples of minority groups are needed.

An AD-GRS based on prior GWAS strongly predicts memory and dementia in NHW in this large cohort. Among NHB, a similar AD-GRS excluding APOE also predicts dementia. Previously documented racial differences in the APOE-dementia association were also seen in this national sample, but differences in relative magnitude are consistent with similar absolute effects.

## Supporting Information

**Table S1.** Beta coefficients from AlzGene used to calculate the genetic risk score for dementia. **Table S2.** Pooled logistic regression models: Odds ratios of dementia probability by genetic risk score stratified by race and controlling for population stratification eigenvectors. **Table S3.** Generalized Linear Regression Models: Regression coefficients for memory score by genetic risk score stratified by race and controlling for population stratification eigenvectors. **Table S4.** Age-Stratified Models: Regression coefficients for memory score by genetic risk score stratified by race and age and controlling for population stratification eigenvectors. **Table S5.** Pooled logistic regression models: Odds ratios of dementia probability by genetic risk score stratified by race and gender. **Table S6.** Generalized Linear Regression Models: Regression coefficients for memory score by genetic risk score stratified by race and gender.
