# Supplementary material for: Validation of a polygenic risk score for dementia in black and white individuals
Source: Brain Behav. 2014 Jul 18;4(5):687–97. doi: 10.1002/brb3.248 (PMC4107377; doi:10.1002/brb3.248)
Supplement: Supplementary file 1 [file brb30004-0687-SD1.docx]

SUPPLEMENT

Supplementary Table 1. Beta coefficients from AlzGene used to calculate the genetic risk score for dementia.

| Gene (SNP) | Beta coefficient (log odds ratio) |
| --- | --- |
| APOE(rs429358 & rs7412) | 0.566 |
| BIN1 (rs744373) | 0.153 |
| CLU (rs11136000) | -0.129 |
| ABCA7 (rs3764650) | 0.206 |
| CR1 (rs3818361) | 0.160 |
| PICALM (rs3851179) | -0.129 |
| MS4A6A (rs610932) | -0.101 |
| CD33 (rs3865444) | -0.113 |
| MS4A4E (rs670139) | 0.076 |
| CD2AP (rs9349407) | 0.111 |

Supplementary Table 2. Pooled logistic regression models: Odds ratios of dementia probability by genetic risk score stratified by race and controlling for population stratification eigenvectors.

|  | All  N=7,690  OR (95% CI) | | NHW  N=6,675  OR (95% CI) | | NHB  N=1,015  OR (95% CI) | | All  N=7,690  OR (95% CI) | |
| --- | --- | --- | --- | --- | --- | --- | --- | --- |
| GRS (0.1 increase) | 1.86  (1.55, 2.23)** |  | 2.25 (1.82, 2.78)** |  | 1.21 (0.90, 1.63) |  | 2.22 (1.80, 2.73)** |  |
| GRS  (no APOE)  (0.1 increase) |  | 1.42 (1.07, 1.87)* |  | 1.35 (0.97, 1.88) |  | 1.53 (0.90, 2.62) |  | 1.34 (0.97, 1.86) |
| Black | 0.33 (0.12, 0.91)* | 0.31 (0.12, 0.80)* |  |  |  |  | 0.54 (0.20, 1.49) | 0.26 (0.08, 0.83)* |
| GRS*Black |  |  |  |  |  |  | 0.57 (0.39, 0.82)* |  |
| GRS  (no APOE) *Black |  |  |  |  |  |  |  | 1.21 (0.64, 2.28) |

All models are additionally adjusted for: age (linear), gender, and year of assessment. * p-value <0.05; ** p-value <0.001

Supplementary Table 3. Generalized Linear Regression Models: Regression coefficients for memory score by genetic risk score stratified by race and controlling for population stratification eigenvectors.

|  | All  N=10,401  β (95% CI) | | NHW  N=8,942  β (95% CI) | | NHB  N=1,459  β (95% CI) | | All  N=  β (95% CI) | |
| --- | --- | --- | --- | --- | --- | --- | --- | --- |
| GRS (0.1 increase) | -0.06  (-0.07,  -0.04)** |  | -0.07  (-0.09,  -0.06)** |  | -0.01  (-0.03, 0.02) |  | -0.08  (-0.09,  -0.06)** |  |
| GRS  (no APOE)  (0.1 increase) |  | -0.27  (-0.51,  -0.04)* |  | -0.03  (-0.06,  -0.01)* |  | 0.001  (-0.05, 0.05) |  | -0.04  (-0.06,  -0.01)* |
| Black |  |  |  |  |  |  | -0.46  (-0.55,  -0.37)** | -0.43  (-0.53,  -0.32)** |
| GRS*Black |  |  |  |  |  |  | 0.08 (0.04, 0.11)** |  |
| GRS  (no APOE) *Black |  |  |  |  |  |  |  | 0.04  (-0.02, 0.10) |

All models are additionally adjusted for: age (linear), gender, and year of assessment. * p-value <0.05; ** p-value <0.001

Supplementary Table 4. Age-Stratified Models: Regression coefficients for memory score by genetic risk score stratified by race and age and controlling for population stratification eigenvectors.

|  | NHW | | | NHB | | |
| --- | --- | --- | --- | --- | --- | --- |
|  | All | Age 50-64 | Age 65+ | All | Age 50-64 | Age 65+ |
|  | N=8,942  β (95% CI) | N=3,319  β (95% CI) | N=6,630  β (95% CI) | N=1,459  β (95% CI) | N=642  β (95% CI) | N=993  β (95% CI) |
| GRS (0.1 increase) | -0.03  (-0.40,  -0.11)** | 0.001  (-0.01, 0.01) | -0.11  (-0.13,  -0.08)** | -0.004  (-0.03, 0.02) | -0.002  (-0.02, 0.02) | -0.02  (-0.06, 0.01) |
| Age 65+ | 0.27  (0.24, 0.30)** |  |  | 0.18  (0.12, 0.24)** |  |  |
| GRS*Age 65+ | -0.08  (-0.10,  -0.05)** |  |  | -0.01  (-0.05, 0.03) |  |  |

All models are additionally adjusted for: age (linear), gender, and year of assessment. * p-value <0.05; ** p-value <0.001

Supplementary Table 5. Pooled logistic regression models: Odds ratios of dementia probability by genetic risk score stratified by race and gender.

|  | NHW  OR (95% CI) | | | | | | NHB  OR (95% CI) | | | | | |
| --- | --- | --- | --- | --- | --- | --- | --- | --- | --- | --- | --- | --- |
|  | All  N=6,675 | | Male  N=2,890 | | Female  N=3,785 | | All  N=1,015 | | Male  N=383 | | Female  N=632 | |
|  | Model A^+^  (AD-GRS) | Model B^++^  (AD-GRS ex APOE) | Model A^+^  (AD-GRS) | Model B^++^  (AD-GRS ex APOE) | Model A^+^  (AD-GRS) | Model B^++^  (AD-GRS ex APOE) | Model A^+^  (AD-GRS) | Model B^++^  (AD-GRS ex APOE) | Model A^+^  (AD-GRS) | Model B^++^  (AD-GRS ex APOE) | Model A^+^  (AD-GRS) | Model B^++^  (AD-GRS ex APOE) |
| GRS (0.1 increase) | 2.23  (1.68, 2.92)** |  | 2.01  (1.48, 2.77)** |  | 2.29  (1.72, 3.10)** |  | 1.20  (0.82, 1.73) |  | 1.62  (1.04, 2.51)* |  | 1.20  (0.82, 1.77) |  |
| GRS  (no APOE)  (0.1 increase) |  | 1.26  (0.80, 1.95) |  | 1.45  (0.94, 2.23) |  | 1.26  (0.79, 1.99) |  | 1.30  (0.66, 2.56) |  | 3.60  (1.62, 8.00)** |  | 1.32  (0.66, 2.66) |
| Male | 0.93  (0.61, 1.49) | 0.74  (0.41, 1.35) |  |  |  |  | 0.55  (0.25, 1.35) | 0.25  (0.06, 0.90)* |  |  |  |  |
| GRS*Male | 1.00  (0.66, 1.52) |  |  |  |  |  | 1.42  (0.78, 2.59) |  |  |  |  |  |
| GRS  (no APOE) *Male |  | 1.23  (0.65, 2.35) |  |  |  |  |  | 3.03  (1.43, 8.94)* |  |  |  |  |

All models are additionally adjusted for: age (linear), gender, and year of assessment. * p-value <0.05; ** p-value <0.001

+ Model A uses the regular 10 locus AD-GRS as a predictor

++ Model B uses the alternative 9 locus AD-GRS excluding APOE as a predictor

Supplementary Table 6. Generalized Linear Regression Models: Regression coefficients for memory score by genetic risk score stratified by race and gender.

|  | NHW  β (95% CI) | | | | | | NHB  β (95% CI) | | | | | |
| --- | --- | --- | --- | --- | --- | --- | --- | --- | --- | --- | --- | --- |
|  | All  N=8,942 | | Male  N=3,759 | | Female  N=5,183 | | All  N=1,459 | | Male  N=522 | | Female  N=937 | |
|  | Model A^+^  (AD-GRS) | Model B^++^  (AD-GRS ex APOE) | Model A^+^  (AD-GRS) | Model B^++^  (AD-GRS ex APOE) | Model A^+^  (AD-GRS) | Model B^++^  (AD-GRS ex APOE) | Model A^+^  (AD-GRS) | Model B^++^  (AD-GRS ex APOE) | Model A^+^  (AD-GRS) | Model B^++^  (AD-GRS ex APOE) | Model A^+^  (AD-GRS) | Model B^++^  (AD-GRS ex APOE) |
| GRS (0.1 increase) | -0.08  (-0.10,  -0.05)** |  | -0.07  (-0.10,  -0.05)** |  | -0.07  (-0.10,  -0.05)** |  | -0.001  (-0.03, 0.03) |  | -0.03  (-0.08,  0.01) |  | -0.001  (-0.03, 0.03) |  |
| GRS  (no APOE)  (0.1 increase) |  | -0.03  (-0.07, 0.01) |  | -0.04  (-0.08,  -0.01)* |  | -0.03  (-0.07,  0.01) |  | 0.02  (-0.05, 0.09) |  | -0.05  (-0.12, 0.02) |  | 0.02  (-0.05, 0.09) |
| Male | -0.19  (-0.22,  -0.15)** | -0.17  (-0.22,  -0.12)** |  |  |  |  | -0.15  (-0.22,  -0.07)* | -0.10  (-0.21, 0.01) |  |  |  |  |
| GRS*Male | 0.01  (-0.03, 0.04) |  |  |  |  |  | -0.03  (-0.08,  -0.03) | -0.07  (-0.16, 0.03) |  |  |  |  |
| GRS  (no APOE) *Male |  | -0.01  (-0.06, 0.04) |  |  |  |  |  |  |  |  |  |  |

All models are additionally adjusted for: age (linear), gender, and year of assessment. * p-value <0.05; ** p-value <0.001

+ Model A uses the regular 10 locus AD-GRS as a predictor

++ Model B uses the alternative 9 locus AD-GRS excluding APOE as a predictor
